# Extramedullary *versus* intramedullary tibial alignment technique in total knee arthroplasty: A meta-analysis of randomized controlled trials

**DOI:** 10.6061/clinics/2015(10)10

**Published:** 2015-10

**Authors:** Huan Bei Zeng, Xiao Zhou Ying, Guang Jun Chen, Xia Qing Yang, Duo Duo Lin, Zhi Jie Li, Hai Xiao Liu

**Affiliations:** Second Affiliated Hospital of Wenzhou Medical University, Department of Orthopaedic Surgery, Wenzhou, China

**Keywords:** Extramedullary, Intramedullary, Total Knee Arthroplasty, Meta-Analysis

## Abstract

The aim of this study was to establish whether the use of an extramedullary or intramedullary tibial cutting guide leads to superior mechanical leg axis and implant positioning. A meta-analysis of six randomized controlled trials including 350 knees was performed. For the mechanical axis, frontal tibial component angle and tibial slope, there were no significant differences in the mean values or the number of outliers (±3°) between the extramedullary and intramedullary groups. A reduced tourniquet time was associated with the intramedullary guide. No significant difference in the complication rate was noted between the two groups. Neither extramedullary nor intramedullary tibial alignment was more accurate in facilitating the tibial cut. Use of an intramedullary guide results in a shorter tourniquet time and exhibits a similar complication rate as the extramedullary guide.

## INTRODUCTION

Total knee arthroplasty (TKA) is a successful procedure for the treatment of pain and for restoring physical function in patients with severe arthritis 1-4. Lower extremity alignment is one of the paramount factors determining the long-term success of TKA. The survival rate of TKA is increased if leg alignment is restored within 3° of valgus or varus on the mechanical axis 5,6. Malpositioning of the implant can lead to early wear and loosening as well as inferior functional performance 7,8, which potentially exposes patients to reduced implant longevity 9-11.

Both intramedullary (IM) and extramedullary (EM) techniques are popular for guiding tibial component alignment in TKA. However, significant debate still exists regarding the optimal alignment guide for the placement of the tibial component. Most TKA systems offer both methods at the choice of the surgeon. Each tibial instrumentation method is reliable, although different authors have presented opposing results with respect to which type of tibial instrumentation results in better component alignment 12-14. Some randomized controlled trials (RCTs) have been published comparing the IM with the EM guide for tibial component alignment in TKA. However, a meta-analysis evaluating the radiographical outcomes between the two guiding techniques has not been performed.

In this study, we conducted a meta-analysis of pooled data from relevant RCTs to evaluate whether an IM or EM tibial guide is more accurate in assuring correct tibial positioning. Moreover, the tourniquet time and complication rate were also compared between these two techniques.

## MATERIALS AND METHODS

**Figure 1:**
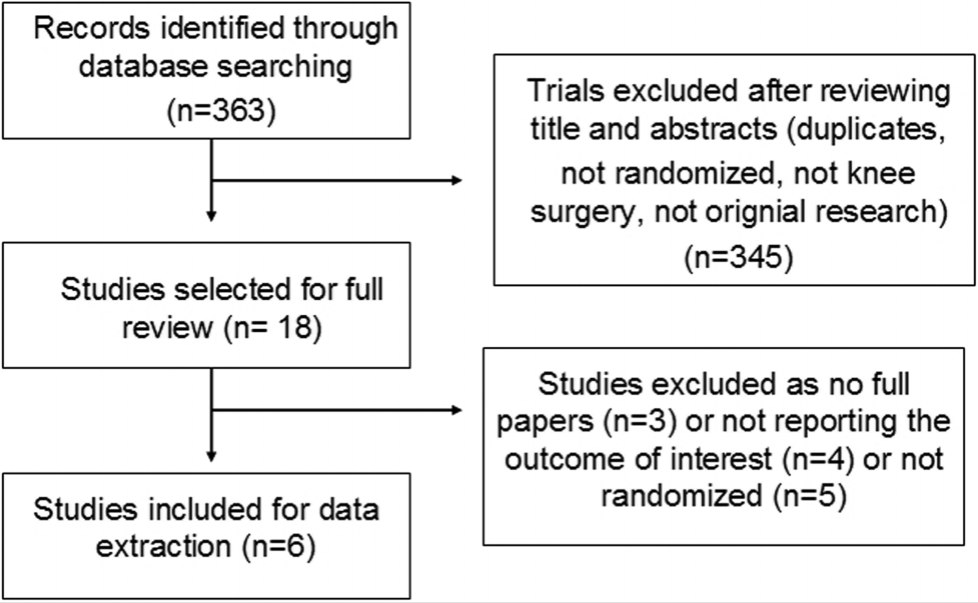
Flow chart of the study selection and inclusion process.

We conducted a meta-analysis of all English and non-English articles identified from electronic databases including Medline, Embase, Cochrane Library, China National Knowledge Infrastructure, Wan Fang Chinese Periodical and Google. In addition, we also manually searched for other relevant studies including those from the reference lists of all included studies. The last search was conducted on September 2, 2014. We used the following key words: arthroplasty, replacement, knee, total knee arthroplasty, randomized, randomised, intramedullary and extramedullary. These key words were used in combination with the Boolean operators AND or OR. The search strategy is presented in Figure 1.

### Exclusion criteria and quality criteria

We included all published RCTs comparing EM guides with IM guides in patients undergoing primary TKA. Exclusion criteria comprised the following: trials with a retrospective design and trials that did not randomize patients into two relevant groups. Quality criteria included the randomization method, concealment of allocation, blinding and intention-to-treat analysis.

### Data extraction

For each eligible study, two of the authors of this meta-analysis independently extracted all relevant data. Disagreement was resolved by discussion with a third investigator. The following data were extracted: 1) the participants' demographic data; 2) the mean value of the mechanical axis and the number of outliers (±3°); 3) the mean value of the frontal tibial component (FTC) angle and the number of outliers (±3°); 4) the mean value of the tibial slope; 5) the tourniquet time; 6) and the complication rate. When data were incomplete or unclear, attempts were made to contact the investigators for clarification.

### Radiologic limb alignment

The mechanical axis was defined as a line bisecting the center of the femoral head, the center of the knee and the center of the ankle. The FTC angle was defined as the angle measured between the articular surface of the tibial component and the mechanical axis of the tibia with a goal of 90°. The tibial slope was measured as the angle between the tibial plateau and a line perpendicular to the anatomical axis of the tibia. Up to 3° of deviation from neutral alignment was considered acceptable, whereas values outside of this range were classified as outliers.

### Data analysis

This meta-analysis was conducted using RevMan 5.0 (Cochrane Collaboration, Oxford, UK). We assessed the statistical heterogeneity using a standard chi-square test (statistical heterogeneity was considered to be present at *p*<0.1 and I^2^ values >50%). When comparing trials exhibiting heterogeneity, pooled data were meta-analyzed using a random effects model; otherwise, a fixed effects model was used. Mean differences and 95% confidence intervals (CIs) were calculated for continuous outcomes and risk ratios (RR) and 95% CIs were calculated for dichotomous outcomes.

## RESULTS

A total of 363 potentially relevant papers were identified. By screening titles and reading the abstracts and the entire articles, six studies with 350 knees (173 in the EM group and 177 in the IM group) were included in the final meta-analysis. All of these RCTs were published in English. The sample sizes ranged from 50 to 100 knees. Five studies assessed radiological alignment 13-18 and one study assessed functional outcomes 19. The key characteristics of the included RCTs are summarized in Table 1.

### Methodological quality

**Figure 2:**
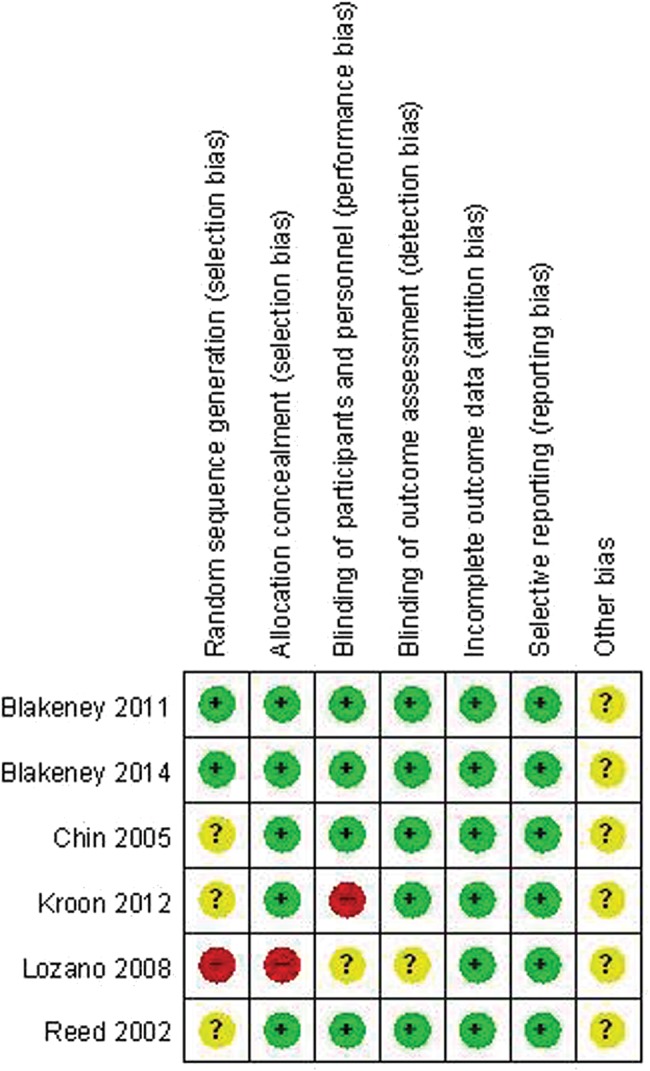
Methodological quality of the included studies. This risk of bias tool incorporates assessment of randomization (sequence generation and allocation concealment), blinding (participants, personnel and outcome assessors), completeness of outcome data, selection of outcomes reported and other sources of bias. The items were scored with “yes”, “no”, or “unsure”.

**Figure 3:**
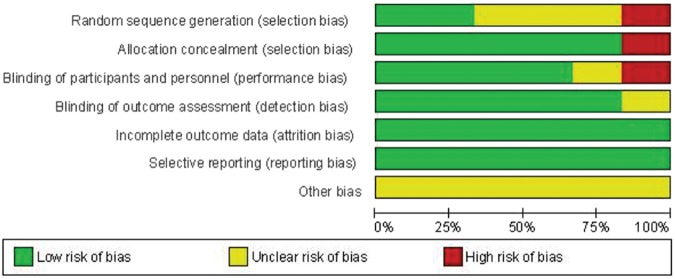
Risk of bias. Each risk of bias item is presented as a percentage across all included studies and indicates the proportional level for each risk of bias item.

The methodological quality of the six included studies was variable. The reported methods of generating allocation sequences were adequate in two studies and five trials reported allocation concealment. Blinding of the surgeon and the patients was reported in four studies and five of the studies blinded their assessors to the outcome. The methodological quality of the studies is presented in Figure 2. Judgment with respect to each risk of bias item is presented as a percentage for all of the included studies, as shown in Figure 3.

### Comparison of radiologic limb alignment

**Figure 4:**
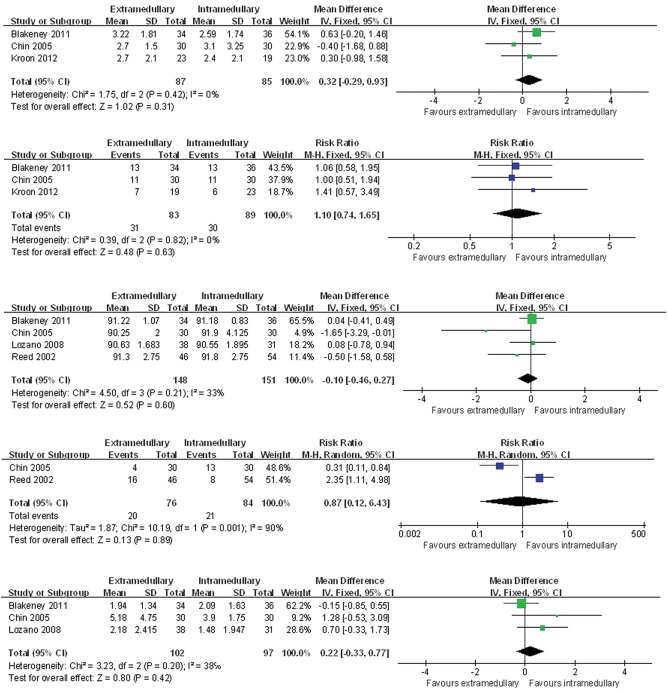
Comparison of the mean mechanical axis between the extramedullary and the intramedullary technique. b. Relative risk of producing a deviation of greater than 3o from neutral in the mechanical axis in extramedullary group vs. the intramedullary group. c. Comparison of the mean FTC angle between the extramedullary and intramedullary technique. d. Relative risk of producing deviation of greater than 3o from neutral in the FTC angle for the extramedullary vs. intramedullary techniques. e. Comparison of the mean tibial slope between the extramedullary and intramedullary groups.

The pooled results indicated that there was no significant difference between the two groups in terms of the mean mechanical axis (*p*=0.31, Figure 4a). Outliers in the mechanical axis occurred in 37.3% of knees (31/83) in the EM group compared with 33.7% (30/89) in the IM group and there was no significant difference (*p*=0.63, Figure 4b). No significant difference in the mean FTC angle was noted between the two groups (*p*=0.60, Figure 4c). The outliers in the FTC angle occurred in 26.3% of knees (20/76) in the EM group compared with 25% (21/84) in the IM group and there was no significant difference (*p*=0.89, Figure 4d). There was no significant difference between the two groups in terms of the mean tibial slope (*p*=0.42, Figure 4e).

### Comparison of the tourniquet time and the complication rate

**Figure 5:**
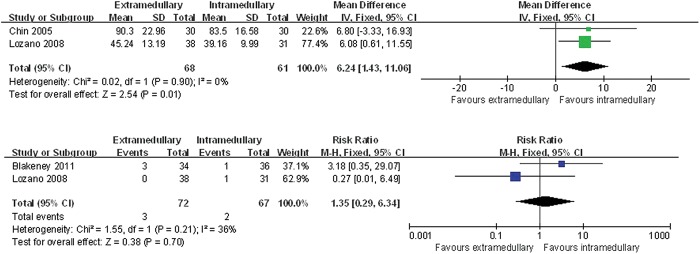
Comparison of the mean tourniquet time between the extramedullary group and the intramedullary group. b. Comparison of the complication rate between the extramedullary group and the intramedullary group.

The tourniquet time was shorter in the IM group compared with the EM group (*p*=0.01, Figure 5a). TKA-related complications occurred in 4.2% of knees (3/72) in the EM group compared with 3.0% (2/67) in the IM group with no significant difference (*p*=0.70, Figure 5b). Blakeney et al. 17 stated that there was one case of pulmonary embolism, one deep infection and one case of knee stiffness in the EM group and one case of knee stiffness in the IM group. Chin et al. 15 reported that one patient in the IM group had a mild stroke. The functional scores were evaluated, although insufficient data were available for the meta-analysis. Only one study measured functional knee scores (Oxford knee score) with mean scores of 37.6 in the EM group and 36.8 in the IM group.

## DISCUSSION

Our meta-analysis compared the radiographic outcomes between the EM and the IM guiding techniques in patients undergoing TKA. No significant differences were found between the two groups in terms of the mean values of the mechanical axis, the FTC angle or the tibial slope. Moreover, neither the EM nor the IM guiding techniques offer an advantage over the other method in reducing outliers of greater than 3°. The IM guide is associated with a shorter tourniquet time and exhibits a similar complication rate as the EM guide. A comparison of functional outcomes between the two groups could not be performed in this study because RCT research on functional outcomes is scarce.

A previous study of British orthopedic surgeons found that 75.6% prefer EM and 20.3% prefer IM jigs when determining tibial alignment with the remainder using both or neither 20. The published literature is divided as to which jig is superior. According to the results of our literature review, approximately 52.6% studies argue that IM and EM guides are equally accurate for tibial alignment 12,16-18,. Approximately 36.8% suggest that an IM guide is more accurate (13,14,27-30,34) and 10.5% suggest that an extramedullary guide is more accurate 31,32. However, very few studies included large samples or RCTs for a comparison of the two methods. Regarding the accuracy of the tibial cutting, this current meta-analysis study suggests that neither EM nor IM tibial alignment is more accurate than the other approach.

Most surgeons prefer to use the extramedullary guide either because they are more experienced in its use or because of the possible complications of the IM guide. However, because the center of the talus is slightly medial to the midpoint between the malleoli, the surgeon must estimate the location of the center of the talus based on these bony landmarks, which may be obscured by soft tissue in obese patients or by bony abnormalities 33. For IM guides, the entry point position is a key factor and the ideal entry point position is located on the tibial articular surface corresponding to the proximal continuation of the tibial canal and should be preoperatively determined with the help of X-rays 34. Moreover, decompression of the medullary contents using suction before instrumenting the canal is also recommended to decrease the risk of embolizing the medullary contents 35. Therefore, it is important for the surgeon to appreciate the benefits and deficiencies of each guide and to use whichever method is most appropriate for each particular case, although both the EM and IM systems allow for satisfactory alignment.

Most surgeons have accepted that a postoperative mechanical axis of 0°±3° will result in less pain, better knee function, faster rehabilitation and improved quality of life 36-39. Recently, several studies found that a postoperative mechanical axis of 0°±3° did not result in better long-term survival of TKA implants compared with a group of outliers 39-43. In one of the most influential studies, Parratte et al. 40 retrospectively reviewed the data of 398 primary TKAs and found that a mechanical axis of 0°±3° did not improve the rate of survival 15 years postoperatively. This result implies that the accuracy of the mechanical axis likely provides limited value with regards to long-term durability. In addition, although computer-assisted TKA improves the mechanical leg axis and component orientation compared with the conventional technique, there is currently no proven clinical benefit of this approach. Therefore, future research on tibial guiding techniques should not only assess radiological alignment but also consider functional outcomes.

This present meta-analysis has several limitations. First, only six studies were included and the sample size of the included studies was small, which might have affected our results. Second, most of the trials focused on short-term radiographic outcomes and only one RCT study evaluated functional outcome. Therefore, we could not perform a valid statistical comparison of the functional outcomes between the two groups. Therefore, further high-quality RCTs with long-term follow-up should be designed to assess radiographic outcomes, knee function and implant survival rate.

## Figures and Tables

**Table 1:** Characteristics of the included studies.

Author	Country	Patients(EM)/(IM)	Age(EM)/(IM)	Gender(EM)/(IM)	Total knee system	Evaluation of alignment
Blakeney 2011 (17) 2014 (19)	Australia	34/36 35/36	<70 years:11/19; >70 years: 23(24)/17	Male:12/15 Female: 23/21	Genesis II total knee system (Smith&Nephew)	6 weeks for X ray; 3 months for CT
Kroon 2012 (18)	Netherlands	24/26	Not stated	Not stated	Genesis II MIS-TKA (Smith&Nephew)	4 to 6 monthsX ray
Lozano 2008 (16)	Spain	38/31	70 years old/ 69 years old	Male:7/3 Female: 32/28	Profix total knee system (Smith&Nephew)	Examination time: Not stated X ray
Chin 2005 (15)	Singapore	30/30	65.6 years old/ 66.9 years old	Male:7/6 Female: 23/24	PFC Sigma total knee system (Depuy)	Examination time: Not stated X ray
Reed 2002 (14)	United Kingdom	46/54	68 years old/ 69 years old	Male: 24/22 Female: 28/26	Not stated	3 months X ray

EM: Extramedullary group; IM: Intramedullary group.

## References

[1] Gandhi R, Dhotar H, Razak F, Tso P, Davey JR, Mahomed NN (2010). Predicting the longer term outcomes of total knee arthroplasty. Knee.

[2] Gioe TJ, Sinner P, Mehle S, Ma W, Killeen KK (2007). Excellent survival of all-polyethylene tibial components in a community joint registry. Clin Orthop Relat Res.

[3] Julin J, Jamsen E, Puolakka T, Konttinen YT, Moilanen T (2010). Younger age increases the risk of early prosthesis failure following primary total knee replacement for osteoarthritis: a follow-up study of 32,019 total knee replacements in the Finnish arthroplasty register. Acta Orthop.

[4] Spencer SJ, Baird K, Young D, Tait GR (2012). The rotaglide mobile bearing knee arthroplast A 10 to13 year review from an independent centre. Knee.

[5] Sikorski JM (2008). Alignment in total knee replacement. J Bone Joint Surg Br.

[6] Ensini A, Catani F, Leardini A, Romagnoli M, Giannini S (2007). Alignments and clinical results in conventional and navigated total knee arthroplasty. Clin Orthop Relat Res.

[7] Berger RA, Crossett LS, Jacobs JJ, Rubash HE (1998). Malrotation causing patellofemoral complications after total knee arthroplasty. Clin Orthop Relat Res.

[8] Jeffery RS, Morris RW, Denham RA (1991). Coronal alignment after total knee replacement. J Bone Joint Surg Br.

[9] Fang DM, Ritter MA, Davis KE (2009). Coronal alignment in total knee arthroplasty: just how important is it. J Arthroplasty.

[10] Incavo SJ, Wild JJ, Coughlin KM, Beynnon BD (2007). Early revision for component malrotation in total knee arthroplasty. Clin Orthop Relat Res.

[11] Sharkey PF, Hozack WJ, Rothman RH, Shastri S, Jacoby SM (2002). Why are total knee arthroplasties failing today. Clin Orthop Relat Res.

[12] Rottman SJ, Dvorkin M, Gold D (2005). Extramedullary versus intramedullary tibial alignment guides for total knee arthroplasty. Orthopedics.

[13] Brys DA, Lombardi AV, Mallory TH, Vaughn BK (1991). A comparison of intramedullary and extramedullary alignment systems for tibial component placement in total knee arthroplasty. Clin Orthop.

[14] Reed MR, Bliss W, Sher JL, Emmerson KP, Jones SM, Partington PF (2002). Extramedullary or intramedullary tibial alignment guides: a randomised, prospective trial of radiological alignment. J Bone Joint Surg Br.

[15] Chin PL, Yang KY, Yeo SJ, Lo NN (2005). Randomized control trial comparing radiographic total knee arthroplasty implant placement using computer navigation versus conventional technique. J Arthroplasty.

[16] Lozano LM, Segur JM, Maculé F, Núñez M, Torner P, Castillo F (2008). Intramedullary versus extramedullary tibial cutting guide in severely obese patients undergoing total knee replacement: a randomized study of 70 patients with body mass index &gt;35 kg/m2. Obes Surg.

[17] Blakeney WG, Khan RJK, Wall SJ (2011). Computer-assisted techniques versus conventional guides for component alignment in total knee arthroplasty: a randomized controlled trial. J Bone Joint Surg Am.

[18] Kroon KE, Houterman S, Janssen RP (2012). Leg alignment and tibial slope after minimal invasive total knee arthroplasty: a prospective, randomized radiological study of intramedullary versus extramedullary tibial instrumentation. The Knee.

[19] Blakeney WG, Khan RJK, Palmer JL (2014). Functional outcomes following total knee arthroplasty: A randomised trial comparing computer-assisted surgery with conventional techniques. The Knee.

[20] Phillips AM, Goddard NJ, Tomlinson JE (1996). Current techniques in total knee replacement: results of a national survey. Ann R Coll Surg Engl.

[21] Confalonieri N, Manzotti A, Pullen C, Ragone V (2005). Computer-assisted technique versus intramedullary and extramedullary alignment systems in total knee replacement: a radiological comparison. Acta Orthop Belg.

[22] Yang SH, Liu TK (1998). Intramedullary versus extramedullary tibial alignment guides in total knee arthroplasty. J Formos Med Assoc.

[23] Ishii Y, Ohmori G, Bechtold JE, Gustilo RB (1995). Extramedullary versus intramedullary alignment guides in total knee arthroplasty. Clin Orthop Relat Res.

[24] Tillett ED, Engh GA, Petersen TA (1988). Comparative study of extramedullary and intramedullary alignment systems in total knee arthroplasty. Clin Orthop Relat Res.

[25] Teter KE, Bregman D, Colwell CW (1995). Accuracy of intramedullary versus extramedullary tibial alignment cutting systems in total knee arthroplasty. Clin Orthop Relat Res.

[26] Mihalko WM, Krackow K (2006). Differences between extramedullary, intramedullary, and computer-aided surgery tibial alignment techniques for total knee arthroplasty. J Knee Surg.

[27] Cashman JP, Carty FL, Synnott K, Kenny PJ (2011). Intramedullary versus extramedullary alignment of the tibial component in the Triathlon knee. J Orthop Surg Res.

[28] Laskin RS (2001). Intramedullary instrumentation: safer and more accurate than extramedullary instrumentation. Orthopedics.

[29] Maestro A, Harwin SF, Sandoval MG, Vaquero DH, Murcia A (1998). Influence of intramedullary versus extramedullary alignment guides on final total knee arthroplasty component position: a radiographic analysis. J Arthroplasty.

[30] Engh GA, Petersen TL (1990). Comparative experience with intramedullary and extramedullary alignment in total knee arthroplasty. J Arthroplasty.

[31] Jessup DE, Worland RL, Clelland C, Arredondo J (1997). Restoration of limb alignment in total knee arthroplasty: evaluation and methods. J South Orthop Assoc.

[32] Dennis DA, ChannerM, Susman MH, Stringer EA (1993). Intramedullary versus extramedullary tibial alignment systems in total knee arthroplasty. J Arthroplasty.

[33] Siston RA, Daub AC, Giori NJ, Goodman SB, Delp SL (2005). Evaluation of methods that locate the center of the ankle for computer-assisted total knee arthroplasty. Clin Orthop Relat Res.

[34] Karade V, Ravi1 B, Agarwal M (2012). Extramedullary versus intramedullary tibial cutting guides in megaprosthetic total knee replacement. J Orthop Surg Res.

[35] Amro RR, Nazarian DG, Norris RB, Kelly MP, Booth RE (2001). Suction instrumentation decreases intramedullary pressure, pulmonary embolism during total knee arthroplasty. Univ Penn Orthop J.

[36] Czurda T, Fennema P, Baumgartner M, Ritschl P (2010). The association between component malalignment and post-operative pain following navigation-assisted total knee arthroplasty: results of a cohort/nested case-control study. Knee Surg Sports Traumatol Arthrosc.

[37] Nicoll D, Rowley DI (2010). Internal rotational error of the tibial component is a major cause of pain after total knee replacement. J Bone Joint Surg Br.

[38] Choong PF, Dowsey MM, Stoney JD (2009). Does accurate anatomical alignment result in better function and quality of life? A prospective randomized controlled trial comparing conventional and computer-assisted total knee arthroplasty. J Arthroplasty.

[39] Longstaff LM, Sloan K, Stamp N, Scaddan M, Beaver R (2009). Good alignment after total knee arthroplasty leads to faster rehabilitation and better function. J Arthroplasty.

[40] Parratte S, Pagnano MW, Trousdale RT, Berry DJ (2010). Effect of postoperative mechanical axis alignment on the fifteen-year survival of modern, cemented total knee replacements. J Bone Joint Surg Am.

[41] Matziolis G, Krocker D, Weiss U, Tohtz S, Perka C (2007). A prospective, randomized study of computer-assisted and conventional total knee arthroplasty. Three-dimensional evaluation of implant alignment and rotation. J Bone Joint Surg Am.

[42] Bonner TJ, Eardley WG, Patterson P, Gregg PJ (2011). The effect of post-operative mechanical axis alignment on the survival of primary total knee replacements after a follow-up of 15 years. J Bone Joint Surg Br.

[43] Morgan SS, Bonshahi A, Pradhan N, Gregory A, Gambhir A, Porter ML (2008). The influence of postoperative coronal alignment on revision surgery in total knee arthroplasty. Int Orthop.

